# Measuring persistence of implementation: QUERI Series

**DOI:** 10.1186/1748-5908-3-21

**Published:** 2008-04-22

**Authors:** Candice C Bowman, Elisa J Sobo, Steven M Asch, Allen L Gifford, the HIV/Hepatitis Quality Enhancement Research Initiative

**Affiliations:** 1 Health Services Research & Development, VA San Diego Healthcare System, San Diego, California, USA; 2 Department of Anthropology, San Diego State University, San Diego, California, USA; 3 Center for the Study of Healthcare Provider Behavior, VA Greater Los Angeles Healthcare System, Los Angeles, California, USA; 4 Center for Health Quality, Outcomes, and Economic Research, VA New England Healthcare System, Bedford, Massachusetts, USA

## Abstract

As more quality improvement programs are implemented to achieve gains in performance, the need to evaluate their lasting effects has become increasingly evident. However, such long-term follow-up evaluations are scarce in healthcare implementation science, being largely relegated to the "need for further research" section of most project write-ups. This article explores the variety of conceptualizations of implementation sustainability, as well as behavioral and organizational factors that influence the maintenance of gains. It highlights the finer points of design considerations and draws on our own experiences with measuring sustainability, framed within the rich theoretical and empirical contributions of others. In addition, recommendations are made for designing sustainability analyses.

This article is one in a *Series *of articles documenting implementation science frameworks and approaches developed by the U.S. Department of Veterans Affairs Quality Enhancement Research Initiative (QUERI).

## Background

When quality improvement (QI) programs reach initial success but fail to maintain it, the need for guidance in evaluating the lasting effects of implementation becomes evident. However, any real measurement of long-term effects is rare and sporadic in the burgeoning discipline of healthcare implementation science. Akin to other aspects of healthcare, such as the pharmaceutical industry's post-marketing phase of pharmacovigilance for monitoring ongoing drug quality and safety, more prospective studies that follow implementation program dynamics over the long term are needed. As things stand, very little is known about what eventually happens to outcomes – or whether new programs even still exist after implementation is completed [1].

This article is one in a *Series *of articles documenting implementation science frameworks and approaches developed by the U.S. Department of Veterans Affairs (VA) Quality Enhancement Research Initiative (QUERI). QUERI is briefly outlined in Table 1 and is described in more detail in previous publications [2,3]. The *Series' *introductory article [4] highlights aspects of QUERI that are related specifically to implementation science, and describes additional types of articles contained in the *Series*. In this case, the focus is on a key measurement issue – sustainability, which is a discrete component of the QUERI model's fourth phase of refinement and spread.

**Table 1:** The VA Quality Enhancement Research Initiative (QUERI)

The U.S. Department of Veterans Affairs' (VA) Quality Enhancement Research Initiative (QUERI) was launched in 1998. QUERI was designed to harness VA's health services research expertise and resources in an ongoing system-wide effort to improve the performance of the VA healthcare system and, thus, quality of care for veterans.

QUERI researchers collaborate with VA policy and practice leaders, clinicians, and operations staff to implement appropriate evidence-based practices into routine clinical care. They work within distinct disease- or condition-specific QUERI Centers and utilize a standard six-step process:

1) Identify high-risk/high-volume diseases or problems.
2) Identify best practices.
3) Define existing practice patterns and outcomes across the VA and current variation from best practices.
4) Identify and implement interventions to promote best practices.
5) Document that best practices improve outcomes.
6) Document that outcomes are associated with improved health-related quality of life.

Within Step 4, QUERI implementation efforts generally follow a sequence of four phases to enable the refinement and spread of effective and sustainable implementation programs across multiple VA medical centers and clinics. The phases include:

1) Single site pilot,
2) Small scale, multi-site implementation trial,
3) Large scale, multi-region implementation trial, and
4) System-wide rollout.

We explore the concept of sustainability and related design considerations in the context of our experiences from a QUERI project, where we sought to measure whether implemented changes were sustained (see Table 2 for project details). Such knowledge is essential to the creation of durable, exportable implementation products that can be broadly rolled-out across the VA healthcare system, an expectation consistent with the QUERI framework.

**Table 2:** QUERI-HIV/Hepatitis implementation project summary

**MAIN IMPLEMENTATION PROJECT:**	
Background:	Although studies have shown that real-time computerized clinical reminders (CR) modestly improve essential chronic disease care processes, no studies have compared the separate and combined effects of CR and group-based quality improvement (GBQI) collaboratives.
Objectives:	To evaluate CR, GBQI, and the interaction of the two in improving HIV quality (Step 4, Phase 2 per the QUERI framework).
Methods:	Using a quasi-experimental design, 4091 patients in 16 VA facilities were stratified into four groups: CR, GBQI, CR+GBQI, and controls. CR facilities received software and technical assistance in implementing real time reminders. GBQI facilities participated in a year-long collaborative emphasizing rapid cycle quality improvement targets of their choice. Ten predefined clinical endpoints included the receipt of highly active antiretroviral (ARV) therapy, screening and prophylaxis for opportunistic infection, as well as monitoring of immune function and viral load. Optimal overall care was defined as receiving all care for which the patient was eligible. Interventional effects were estimated using clustered logistic regression, controlling for clinical and facility characteristics. Human subjects' protection approval was obtained.
Results:	Compared to controls, CR facilities improved the likelihood of hepatitis A, toxoplasma, and lipid screening. GBQI alone improved the likelihood of pneumocystis pneumonia prophylaxis, immune-monitoring on ARVs – but reduced the likelihood of hepatitis B screening. CR+GBQI facilities improved hepatitis A and toxoplasma screening, as well as immune-monitoring on ARV. CR+GBQI facilities improved the proportion of patients receiving optimal overall care (OR = 2.65; CI: 1.16–6.0), while either modality alone did not.
Conclusions:	The effectiveness of CR and GBQI interventions varied by endpoint. The combination of the two interventions was effective in improving overall optimal care quality.

**SUSTAINABILITY ANALYSIS SUPPLEMENT:**	

Objectives:	To ascertain whether the implemented interventions were sustained and became part of routine care, we measured the original outcomes for one additional year and evaluated continued intervention use at selected sites.
Methods:	Interviews with key informants selected from the study sites revealed that some sites had ceased using the interventions, and some control sites had adopted them; analyzing odds of patients receiving guideline-based HIV care (HIVGBC) compared to controls no longer made sense. Thus, we evaluated sustained performance as follows: At the facility-rather than the arm-level, we examined raw rates of patients receiving HIVGBC at only those facilities in the intervention arms that had significant effects in the study year to determine whether they continued to show a significant increase in these rates in the following year, compared to their raw rate at baseline. We also conducted a qualitative component. Based on formative evaluation results assessing the use and usefulness of the reminders, we asked informants if identified barriers were subsequently removed and recommendations heeded. Also, we evaluated the extent to which staff members from the sites that participated in the collaboratives were still conducting rapid-cycle improvement methods to address local care quality problems; whether they still maintained the social networks established during the original study, and the degree to which they were used to disseminate subsequent quality improvement change ideas, and shared network contacts with – and taught the method to new staff.
Results:	For hepatitis A screening, we found that 4 out of the 5 sites that showed a significant increase in their raw rate at 12 months, also showed a significant increase in their raw rate at 24 months compared to baseline (p = .05). For the other four significant indicators of HIVGBC (hepatitis C and toxoplasma screening, CD4/viral load and lipids monitoring), all sites that showed significant increases in their raw performance rates at 12 months, showed a significant increase in their raw rates at 24 months compared to baseline.
Conclusions:	Intervention effects were sustained for one year at nearly all the sites that showed significant increases in performance during the study period. Nearly all sites exposed to reminders were using at least some of the 10 available in the follow-up year. Collaborative methods were still being used, but only at the most activated of the original study sites.

### HIV/Hepatitis QUERI Center project

In a complex and lengthy implementation project, we compared the separate and combined effects of real-time, computerized clinical reminders and group-based quality improvement collaboratives at 16 U.S. Department of Veterans Affairs (VA) healthcare facilities for one year, in order to evaluate each intervention, as well as the interaction of the two, in improving HIV care quality [5]. Then, to ascertain whether performance gains associated with the implemented interventions were sustained and whether or not they had become part of routine care (i.e., had been 'routinized'), we sought guidance from the related literature about how to measure ongoing performance and continued use of the interventions during a follow-up year.

Questions regarding sustaining QI have been addressed in existing work (e.g., Greenhalgh et al. [6], Fixsen et al. [7], and Øvretveit [8]); however, we failed to find enough detail in this literature to actually direct the design and conduct of a sustainability analysis. Therefore, in this article we strive to provide such direction. First, we briefly explore what is known about the lasting effects of quality improvement interventions in regard to long-term behavior change, as well as knowing why gains achieved in performance often fail after implementation. We then provide guidance for implementers interested in measuring not just whether QI changes were likely to sustain, but whether they actually did. We describe important considerations for designing such an analysis, drawing from our own effort, and also framed within the rich theoretical and empirical contributions of others.

### The concept of sustainability

Simply describing implemented interventions as successes or failures leaves too much room for interpretation: What is 'success?' What is 'failure?' What is the timeline by which such conclusions are drawn?

A first step to correcting these ambiguities is making a strong distinction between *achieving *improvement in outcomes and *sustaining *them. Achieving improvements generally refers to gains made during the implementation phase of a project that typically provides a generous supply of support for the intervention, in the way of personnel and other resources. However, sustaining improvements refers to "holding the gains" [p.7, [8]] for a variably defined period after the funding has ceased and project personnel have been withdrawn – an expectation identified as a major challenge to the longevity of public health programs [9]. For this reason, it behooves implementation scientists to keep the longer view in mind when designing interventions, including those that potentially will be exported to other venues.

To paraphrase Fixsen and colleagues, the goal of sustainability is not only the long-term survival of project-related changes, but also continued effectiveness and capacity to adapt or replace interventions or programs within contexts that constantly change [7]. Thus, sustainability also can refer to embedding practices within an organization [6,10-14]. Failure to do so can be a result of either a poor climate for implementation or poor commitment by users because of a misfit with local values [15].

For measurement purposes, this multi-faceted definition needs to be understood and sustainability addressed early in a project. Such an assessment would be unlikely without a proper formative evaluation (FE) built into the original study design [16], as discussed below. This includes assessment of relapse. Although some degree of backsliding happens in any attempt to change behavior, relapsing to old behaviors should be accounted for [10,11]. From a measurement perspective, a decision has to be made regarding how much relapse can be tolerated and still allow investigators to call the new behavior sustained.

As the mention of backsliding suggests, sustainability, as we define it, differs from – but may depend upon – replication when the innovation is expected to spread to additional units or sites within an organization. Similar to fidelity, replication is concerned with how well an innovation stays true to the original validated intervention model after being spread to different cohorts [17]. [We use 'validated' not to indicate testing in highly controlled settings, but rather in regard to testing in more typical, real-time circumstances.] Racine argues that sustained effects can be achieved only through the reliable spread of an innovation, or the faithful replication of it [18].

Slippage of the intervention from the original achievement of change related to its core elements can occur as a result of influences from multiple levels of the organization, due to, for example, local staffing conditions, administrators losing interest, or changes in organizational goals. Slippage can be limited through performance monitoring, standards enforcement, and creating receptive subcultures [19], all strategies requiring some degree of infrastructure support. Yet, shortage of such support is precisely why many QI interventions eventually disappear.

There are four prerequisites for realizing program benefits over time: 1) adequate financial resources, 2) clear responsibility and capacity for maintenance, 3) an intervention that will not overburden the system in maintenance requirements [20], and 4) an intervention that fits the implementing culture and variations of the patient population [21]. Skeptics may argue that these organizational components rarely coincide in the real world. Some see standardizing QI interventions as a pitfall that should be avoided, while some seem less worried about the negative effects of variation because they see it as a necessary adaptation to local environments [22,23]. Preventing adaptation at this level may explain why an intervention did or did not sustain, so imposing fidelity can be a double-edged sword. This can be better understood and clarified by measuring it through both the project's FE and, later, in the follow-up analysis.

To construct an informative definition of sustainability, it is important to keep in mind the fundamental objective of implementation and QI: To improve patient health. For example, effectiveness measured by number of smoking quits or higher screening rates for at-risk patients are typical of implementation project objectives; actual improvements in morbidity and mortality are the inferred endpoints of interest. The overarching concern of any QI effort should be for an intervention or program that survives long enough to lead to improvements in patient health that can be measured. That being said, establishing the relationship between a particular QI strategy and its related health outcome(s) may be somewhat ambitious considering the barriers [24].

Following Øvretveit [8], we conceptually define sustainability in two ways: 1) continued use of the core elements of the interventions, and 2) persistence of improved performance. Operationally defining and measuring 'continued use,' 'core elements,' 'persistence,' and 'improved performance' was a challenge for us – an experience that formed the basis of this article.

### Factors affecting sustainability and its measurement

Spreading innovations within service organizations can range from "letting it happen" to "making it happen," depending on the degree of involvement by stakeholders [6]. The former passive mode is unprogrammed, uncertain and unpredictable, whereas, the latter active mode is planned, regulated and managed [25].

**Figure 1:**
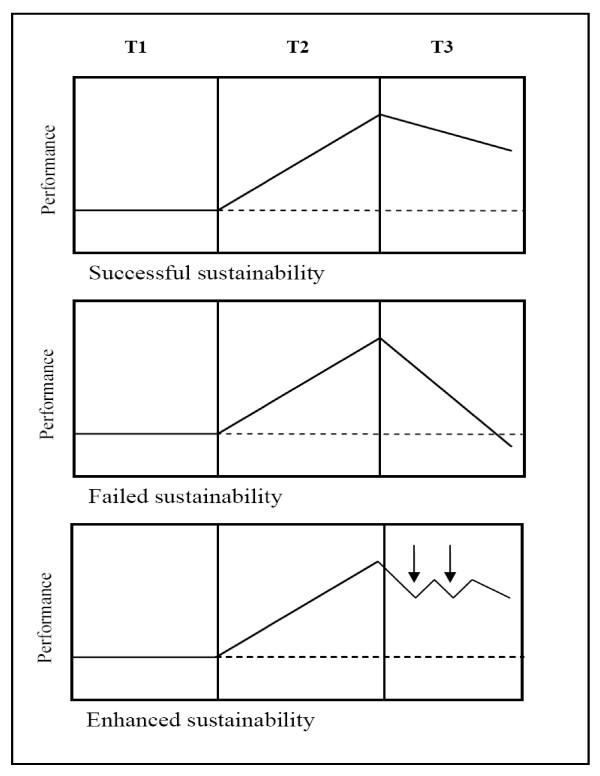
**Three possible outcomes of performance improvement: Successful, failed, and enhanced.** (T1 = baseline period, T2 = implementation period, T3 = follow-up period).

Consider the three situations in Figure 1. In each, performance improvements, shown on the vertical axes, decline when the active phase ends although each ultimately represents a different outcome. In the first situation, the active phase shows initial gains (i.e., *sustainability success*) but performance still decays somewhat when support is withdrawn. However, given a sufficiently receptive environment, improvements can remain above the baseline level in the long-term. Alternatively, the second curve shows performance returning to baseline, and in the worst case, dropping below it, indicating *sustainability failure*. Although either the first or second circumstance could result from a situation where support for the intervention is relatively passive, active maintenance may make all the difference in long-term success; and sustainability failure seems particularly likely if the level of support provided for the intervention during implementation is withdrawn after its completion (e.g., enriched clinic staff, research assistants, leadership endorsement).

In the final situation, which clearly reflects an active mode, a successfully implemented intervention with follow-up booster activity at certain intervals sustains performance improvements, albeit in a somewhat saw-tooth pattern. Declines are attenuated as a result of the periodic nudge, as without occasional reinforcement or higher order structural changes to encourage institutionalization of the new 'steady state,' the new behavior will eventually decay or revert back to its previous state.

In identifying a need for a better model of the maintenance process in health behavior change, Orleans argued that more effort should be focused on maintenance promotion rather than on relapse prevention [26]. Yet, how much do we really know about what is needed to prevent slips and relapses from occurring until an intervention with its associated performance gains is institutionalized? This question cannot begin to be answered until one knows whether implemented change has actually succeeded or failed in the longer term, and to know that, it has to be measured.

Being cognizant of models describing human behavior and lifestyle change may benefit the selection of suitable sustainability measures to study the behavior of providers and organizations. Models most frequently used are based on assumptions that people are motivated by reason or logic, albeit within contexts influenced by social norms or beliefs (e.g., Health Beliefs Model [27] and Theory of Reasoned Action [28]). However, logic is not always a primary driver of behavior [29] but when it is, the logic may be generated in regard to cultural or structural factors (i.e., peer norms or an individual's relationship with his/her supervisor). Measures that capture information about what drives continued participation in QI efforts would be useful.

While change through the internalization of new processes is essential for sustaining implementation, one factor that we have observed to be associated with sustainability failure is a lack of systems-thinking. That is, to capitalize on gains made during the active phase, and to design proper sustainability measures, one must view organizations as complex, adaptive systems [30]. In such systems, processes that promise to be inherently (albeit unintentionally) supportive of the anticipated change can be leveraged with careful planning to both generate and maintain a QI change. Because routinization of innovations drives sustainability, measures should take into consideration the degree to which a given practice has been woven into standard practice at each study site, such as its centrality in the daily process flow and its location in the practice models held and adhered to by personnel [31,32] (e.g., the Level of Institutionalization instrument or LoIn [12]).

### Measuring sustainability as persistence

In seeking examples of studies that included any degree of follow-up evaluation, we found that evaluation of health promotion programs, primarily in regard to improving individual behavior, and continued concordance with treatment guidelines after implementation or dissemination, targeting either provider or organizational performance, were the two most common foci. This is similar to what Greenhalgh's group found in searching for reports on diffusion of service innovations [6]. Overall, however, our impression, like others' [6], was that reported analyses of sustained effects are rare (see Table 3 for examples from our search). We speculate that this scarcity is due to one or more of the following reasons.

**Table 3:** Examples of studies reporting follow-up evaluation of implemented interventions

**Authors**	**Design**	**Intervention**	**Intervention target**	**Intervention length**	**Outcome measured**	**Post-intervention sustainability period**
Knox et al., 2003 [46]	Quasi-experiment, pre-post comparison	Multi-component suicide prevention program	USAF personnel (patient-level)	1 year	Relative suicide risk factor rates	1 year
Harland et al., 1999 [47]	RCT, pre-post comparison	1–6 motivational interviews, with or without financial incentive	General medicine practice patients (patient-level)	3 months	Self-reported physical activity	1 year
Shye et al., 2004 [48]	Multi-faceted intervention trial, pre-post comparison	(1) Basic strategy: guideline, education, clinical supports (2) Augmented strategy: basic program with social worker added	HMO PCPs (provider-level)	10 months	Rates of female patients who asked about domestic violence	3 months
Sanci et al., 2000, 2005 [49,50]	RCT, pre-post comparison	Multi-faceted adolescent health education program	General medicine practice physicians (provider-level)	3 months	Observer ratings of skills, self-perceived competency, tested knowledge	4 months, 10 months, 5 years
Perlstein et al., 2000 [51]	Pre-post comparison	Implemented bronchiolitis care guideline	Pediatricians who cared for infants 0–1 year hospitalized with bronchiolitis (provider-level)	--	Patient volumes, length of stay, use of ancillary resources	3 years
Brand et al., 2005 [52]	Program evaluation	COPD management guideline	Hospital physicians (provider-level)	--	Guideline concordance, attitudes and barriers to guidelines, access to available guidelines	2 years
Morgenstern et al., 2003 [53]	Quasi-experiment, pre-post comparison	Multi-component acute stroke treatment education program	Community laypersons (patient-level); Community- and ED-based physicians and EMS responders (provider-level); Stroke care policies (organization-level)	15 months	Number of acute stroke patients who received intravenous tissue plasminogen activator	6 months
Bere et al., 2006 [54]	Controlled trial, pre-post comparison	(1) Fruit and vegetable education program (2) No-cost access to school fruit program	School-age students (patient-level)	1 year	All-day fruit and vegetable intake	1 year
Shepherd et al., 2000 [55]	Systematic review of controlled comparisons with pre-post analysis	Health education interventions that promote sexual risk reduction in women	Sexually active women in any setting, treated by any provider type (patient-level)	Varied from 1 day to 3 years (most lasted 1 to 3 months)	Behavioral outcomes (e.g, condom use, fewer partners, or abstinence, fewer STDs)	Varied from 1 month to 6 months (most were up to 3 months)

Note. To identify studies that measured sustainability, we searched PubMed for reports that included any follow-up analyses of interventions or program effects, using keyword searches for terms such as 'sustain,' 'sustained,' 'sustainability,' and 'follow-up.'

• Since there must be a time gap between when a QI study ends and when sustainability can be appropriately measured, finding a suitable funding mechanism can be a challenge.

• An analysis of sustainability, especially if designed post hoc, is limited in what can be evaluated.

• Good measures of sustainability are not common and/or not immediately obvious, depending on the clarity of one's operational definition of sustainability.

We differentiate between sustainability analyses that are premeditated (e.g., included in an implementation project's formative evaluation and those designed after the fact. The fundamental difference between the two is that the former is limited to measuring the likelihood that changes *will sustain*, as the project will end before the maintenance period occurs. There are several measures that could be included in an FE that would elucidate and potentially enhance realization of this concept [16]. The latter type of analysis is limited to measuring whether use of the intervention or performance gains *actually did sustain *without an ability to influence the probability of that occurrence. The LoIn instrument [12] is perhaps the single best example of this latter approach.

With enough forethought and funding, using both premeditated and post hoc approaches would be optimal. However, without the value of forethought we conducted a post hoc-designed evaluation and, in doing so, realized how uncertain we were about what to measure, when and how to measure it, and how to get funded to do so. What follows is a synthesis of what we learned from our own observations and what useful insights we gleaned from the relevant literature about designing a sustainability analysis of either type.

### What to measure

The goal of QUERI implementation is to create effective innovations that remain robust and sustainable under a dynamic set of circumstances and thus ensure continued reduction in the gaps between best and current practices associated with patient outcomes. Hence, knowing precisely what has been sustained becomes important from a performance and thus measurement perspective – and therein lies the challenge. Relative to long-term effectiveness, what is it about the intervention or program that should survive? And what about the organizational context needs to be understood in order to adequately interpret what the results mean? The following provide one set of dimensions of improvements that can potentially be turned into critical measures of sustainability, depending on whether the analysis is part of a project FE or conducted post hoc. These dimensions include: 1) intervention fit, 2) intervention fidelity, 3) intervention dose, and 4) level of the intervention target.

#### Intervention fit

Effective interventions targeting any level of the organization are not necessarily enduringly useful ones. There is good evidence showing that interventions that are not carefully adapted to the local context will not endure [6]. Sullivan and colleagues described a 'bottom-up' approach using provider input to design a QI program that increased provider buy-in and, hence, sustainability of the intervention [33]. When improvements fail to persist, the researcher's challenge is in drawing the right conclusion about whether the intervention failed because of external influences that occurred after the intervention period, or because it was not considered to be all that useful to the organization in the long-run [15].

In our study, we suspected that sites that were marginally enthusiastic about participating in the modified collaborative may have felt obliged to participate for the sake of the project, but in actuality, failed to perceive any benefit for the long-term. Modest enthusiasm most likely explains some of the poor performance observed. Collaborative participation, as part of our strategy for continuous quality improvement (CQI), could not – and did not for some in our project – work with such an attenuated level of interest. More diagnostic FE [16] might have enhanced our intervention mapping to identify and address this issue early on. ('Intervention mapping' is a borrowed term that describes the development of health promotion interventions and refers to a nonlinear, recursive path from recognition of a problem or gap to the identification of a solution [34].

Implemented interventions generally consist of multiple components, some of which do not demonstrate success. Solberg et al. found that bundling guidelines into a single clinical recommendation is more acceptable to the providers who are meant to follow them [35]. However, in the reminder study arm, we implemented a clinical reminder package that consisted of automated provider alerts for 10 separate aspects of evidence-based HIV treatment. Despite the incomplete success of the full software package (i.e., all the alerts in the package did not generate improvements) it was rolled out intact based on a policy decision. Since sustainability was not an issue for the reminders in the package that had failed to produce significant performance improvements during the project, we simply did not analyze their individual effects in the quantitative aspect of the follow-up sustainability assessment. During interviews with key site-based informants after the study was completed, we learned that some of the implemented reminders were not regarded as helpful by local clinicians in making treatment decisions, which helped us explain their failure to produce significant improvements. In more complex, multi-faceted QI interventions, components should not be so inextricably linked, so that independent evaluation of successful ones is still possible.

#### Intervention fidelity

Because of the general complexity of implementation interventions, it is important to evaluate the discrepancy between what the intervention was like in the original implementation versus what it becomes when sustainability is subsequently measured. Inability to unequivocally credit improvements to a particular intervention within a complex improvement strategy is a common shortcoming of QI research [1]. 'Outcome attribution failure' can be a major, and sometimes insoluble, problem in this type of analysis, making it imperative to fully grasp how an intervention morphs with each new implementation unit, at each new site or new phase of roll-out. Wheeler [36] recalls the contributions of Shewhart's 1931 report on controlling variation in product quality in the manufacturing industry. An important message from his work is that normal variations should be differentiated from those that have 'assignable causes,' which create important but undesirable changes in the product over time.

Although all components of the reminder package were offered to all VA sites after our project's completion, we made no attempt to control local drift in the software or its usage. This could have clouded interpretation of performance in the sustainability period, if we had not interviewed key informants about which specific reminders were still being used and under what circumstances. While quantifying the amount of drift in the way that the reminders were being used would have been preferable, we were at least able to describe variation that occurred based on local need or preference.

The QUERI framework recommends enhancing and sustaining uptake with ongoing or periodic monitoring at the local level to lower the fidelity gap, because rarely is the first iteration of an intervention perfect. Local adaptations/variations are not, within the caveat of staying true to the actual basis of the targeted best practice, anathema to the four-phased QUERI model, especially if they are designed on an appropriate rationale to actually improve goal achievement. However, it is clear that changing circumstances within the organizational environment can be a significant threat to sustainability. Implementation researchers could use more guidance about how to distinguish between the core features of an intervention that should not be allowed to drift, and those features that can be adapted. In any event, understanding how an innovation may have been adapted over time, or why it was discontinued are both important to assess when trying to determine the black box of implementation and its ongoing effects, especially during the early stages of a phased roll-out when refinements can, and should, be made. Therefore, understanding and implementing desirable changes to an intervention should be part of the overall implementation strategy.

#### Intervention dose

The longevity of an adopted intervention may be a direct function of original implementation intensity. The twin concepts of 'dose delivered' and 'dose received', referring to the amount of exposure to and uptake of an implementation intervention [37], provide a focus for related measurement, although only the latter is important to a post hoc-designed analysis. To measure the dose received in a post hoc analysis, a researcher would ask what proportion of the intervention was still being used by the intended targets. After we implemented the study collaborative in our project, a national VA HIV collaborative was conducted during the follow-up year. Based on key informant interviews, sites in the study collaborative that subsequently participated in the national collaborative seemed more enthusiastic about continuing to use collaborative strategies for continuous quality improvement. For example, in terms of the use of social networks for sharing QI information and usage of Plan-Do-Study-Act (PDSA) cycles to address new quality problems. We saw this as a clear indication of dose response, which makes a case for incorporating measurement of any booster activity, either prospectively as part of the project's FE, or retrospectively as part of the sustainability analysis.

#### Intervention target

Measuring sustainability depends on the change that is targeted and whether the focus of the intervention is the individual (i.e., provider or patient) or the organization (i.e., facility or integrated healthcare system), or both. Implementing change at one level while taking into consideration the context of the others (e.g., individual versus group, facility, or system), will produce the most long-lasting impact [38]. Effecting changes in individual versus organizational performance are qualitatively different tasks that require not only different instruments for measuring changes, but acquisition of in-depth knowledge of the processes that control adoption or assimilation of the innovation at either level [6]. Activities associated with the project's FE, such as assessment of facilitators and barriers, will facilitate this latter aspect.

Our implementation targets were individual providers who operate within the small HIV clinic environment at each VA medical center. However, buy-in and support from clinic leadership was an obvious factor impacting implementation effectiveness of both system interventions [39]. Our sustainability analysis did not include a repeat of this assessment, but, in hindsight, its inclusion would have facilitated our interpretation of performance and usage in our follow-up. Just as targeting levels of individual and organizational behavior provide an important framework to maximize success of implementation efforts, being mindful of what happens at multiple levels after the project also applies to measuring success of sustained QI.

Sustainability measurement should flow logically from and within the overall project evaluation, thus highlighting the limitations of our post hoc type of approach. However, measuring some of these critical dimensions as part of the project's FE will go a long way toward explaining why an intervention failed or succeeded in the long-run.

### When to measure

The amount of time that is necessary to follow intervention effects and/or usage is quite variable. In the examples listed in Table 3, evaluations lasted anywhere from several months after implementation to several years. Ideally, one would keep measuring performance – and contextual events – until decline shows some indication of stabilizing or the intervention is no longer useful. Some may argue that the more astute investigator will specify the length of the follow-up period based on a theoretical perspective because then, if the follow-up plan becomes altered, they can at least appeal to theory to assess confidence in their results. Because we were limited by funding in our own analysis, the follow-up period was artificially restricted to one year past the end of active implementation. Another difficulty in timing sustainability measurement, described by Titler [40], is knowing where to draw the line between the implementation and follow-up periods. Distinguishing between lingering improvements from the implementation and true persistence of effects from institutionalization also is a challenge [40].

**Figure 2:**
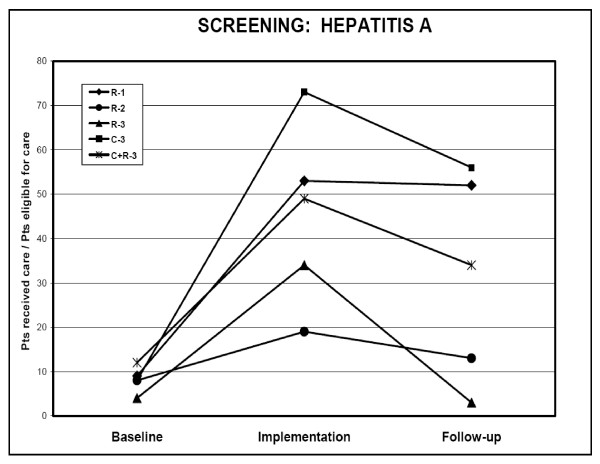
**Site-level performance for hepatitis A screening in HIV patients before, during and after a one-year-implementation trial.** Indicated by letter and number, sites implemented either clinical reminders (R), collaboratives (C), or both (C+R).

In our post hoc analysis, we examined rates of indicated care received by eligible patients at sites within each intervention arm that yielded a significant performance gain during the study year, in order to determine whether that gain persisted in the following year. Figure 2 shows actual performance measurements from our study for appropriate hepatitis A screening in patients with HIV in three time periods: 1) 12 months prior to launch, 2) during the year-long implementation, and 3) during 12 months of follow-up. Judging from the downward trends in screening performance, we probably did not capture the performance nadir, so one year was probably not enough. Our decision to use a one-year follow-up was admittedly arbitrary and based somewhat on symmetry, given that the two earlier periods also were that length. Ideally, the evaluation period should be long enough to capture the lowest level to which performance will naturally decline, either with or without booster efforts to determine success or failure.

The amount of time it takes to measure long-term effects is dependent on the speed of spread [6]. QUERI researchers are not always actively involved in exporting successful QI strategies to other parts of the VA system, thereby making it difficult to know the level of penetration when follow-up evaluation is conducted. Yet due to our close collaboration with the VA central office responsible for HIV care delivery, we did have access to information regarding the level of penetration of our study interventions. Knowing what was happening in the field enabled us to gauge the effects of external events, such as the timing in regard to readiness for subsequent exportation of the reminder software, as well as the schedule for the national collaborative. Then again, there is the temptation for some to use unanticipated external events to excuse a failed intervention. Sustainable QI should be robust to these influences.

CQI methodology dictates that internal follow-up measurement is needed as long as the intervention or program remains useful to the organization [41]. Adding to that, our recommendation for estimating how long to follow performance after implementation would be that 'longer is always better,' although what is feasible rightfully tends to override any other consideration. In translating tenets of CQI to the four-phased QUERI implementation model described in Table 2, the principle remains the same. However, the responsibility for follow-up changes at each phase, such that a move toward creating routine national performance measures should be considered at national roll-out.

### How to measure

Methods used in evaluating the success of implemented QI interventions and strategies are 'messy' at best, and measuring their longer-term effects is no different. A number of designs have been recommended. Focused audit studies have a built-in cycle for monitoring QI impact against accepted and expected standards over time [42]. Other approaches include single case studies and quasi-experimental pre/post-test comparisons [43]. We used the latter design to evaluate the implementation of the clinical reminders and collaborative, although this type of design presented our major challenge in evaluating intervention sustainability for two reasons. First, the non-intervention sites became contaminated after the initial study period because they were allowed to adopt the reminders and participate in the national collaborative, thus preventing any further utility as a comparison group. Second, this snapshot-type of approach prevented a more finely grained examination of when the level of decay might have warranted booster treatments.

Although the lack of an effective control group was not a problem that we could remedy, a more robust approach would have been to use a time-series design [44]. Ideally, the analytic window would have included monthly measurements over the entire 24-month period, while restricting spread of the interventions to the control sites during that time. In hindsight, multiple measurements during the follow-up phase at the very least would have allowed us to take advantage of this potentially useful information.

Because our follow-up analysis was not included as a component of the original design, our default post hoc strategy was to compare rates of patients receiving evidence-based HIV care at only those facilities in the intervention arms that had significant effects in the study year relative to their own rates at baseline. Unfortunately, this approach kept us from associating durability of the effects with a particular intervention, as well as from making site-to-site comparisons. As a result, we were unable to conduct multivariate analyses, although we were able to assess whether improved performance for sites that showed success on certain quality indicators during implementation did persist past the study period. However, finding the right approach to long-term evaluation, given the limits imposed by lack of resources constricting a system's desire to adopt promising interventions, can be a significant barrier to forming valid conclusions about sustainability.

Quantitative methods, however complex, are best suited for measuring ongoing performance, but evaluating implementation interventions or program/strategy usage requires methods that yield more texture and detail (i.e., observation and interviews). May and colleagues evaluated the sustainability of telemedicine services in three projects over five years using such qualitative techniques, enabling them to better determine the how and the why of their empirical results [14]. Similarly, in addition to measuring clinical endpoints, we conducted semi-structured telephone interviews with two key informants from each of the sites that we felt best characterized a particular type of site from each intervention arm. For example, a site that participated in the study collaborative and the national collaborative was chosen, as well as one that participated in the study collaborative only. Answers to questions regarding the existence of known barriers to reminder use and continuation of collaborative activities enhanced our ability to interpret the quantified results.

Implementation barriers and facilitators identified during a project's FE also should be taken into account in designing follow-up sustainability analyses. Based on one component of our project's FE regarding the use and usefulness of the reminders [45], we asked our informants during follow-up if the barriers described in the human factors analysis were subsequently removed and recommendations for procedural modifications heeded. For those sites where barriers had been removed, we were able to conclude that observed performance during the follow-up phase was not a result of the persistence of those barriers.

### How to get funded

Finding financial support to conduct an evaluation of sustained effects will more than likely be a high hurdle for the action-oriented implementation researcher, at least within the U.S., since the traditional three-year grant is rapidly giving way to a shorter and leaner version that obviates any follow-up analyses. Justifying the addition of a fallow period after completion of a project would have been difficult before – and now is virtually impossible without a funded extension. Funded extensions for research grants are rare, although small grants for focused, short-term projects are becoming more common. Such small grants are sufficient to conduct a brief sustainability analysis, as long as the scope is limited and the plan for the evaluation and human subjects' approval is already in place. One caveat for pursuing rapid turnaround grants, such as the one that funded our analysis, is that they may limit the latitude of a qualitative component, in particular, since those analyses are generally more time and resource consuming.

Another option is to entrust the measurement to others. There is a role for the implementation researcher in encouraging participating clinical entities to take on the tasks of monitoring and assessing their own performance. For this to happen, the enterprise needs to make sense to them (i.e., information generated should be useful to their decision making). This is different from researchers who monitor and assess for the sake of generating results for, say, disseminating them to a broader scientific audience. Clinicians need to grasp the value of evaluation as part of their usual practice, so that they understand the importance of having data on important aspects of care delivery that serve the purpose of sustaining change.

## Summary

In this paper, we have summarized the concept of sustainability by briefly reviewing how it has been characterized by others, as well as what factors may affect it from organizational and behavioral perspectives. We defined sustainability as the *continued use of core elements of an intervention and persistent gains in performance as a result of those interventions*, which highlights the distinction made between measuring the potential to sustain as part of an implementation project's FE, and measuring whether usage and improved performance persisted after implementation is completed and the project resources are withdrawn. Finally, we made a number of recommendations regarding the design of a sustainability analysis, which are summarized in Table 4.

**Table 4:** Recommendations for designing a sustainability evaluation

**WHAT TO MEASURE:**
• Don't measure sustainability of interventions that were not useful or didn't achieve a credible level of success.
• Know what particular components of the intervention were actually implemented and/or adapted, and measure sustainability from both a process and outcome point of view.
• Understand the assignable causes of sustainability failure and success.

**WHEN TO MEASURE IT:**

• Allow enough time for performance to decline to its nadir.
• The longer the follow-up period, the better.

**HOW TO MEASURE IT:**

• Build in a follow-up evaluation into the original analytic plan to avoid later challenges, if possible.
• Use more than one method to triangulate qualitative information with quantitative data information.
• Talk directly to local stakeholders to understand the how and why behind
• performance measurements.
• Beware of drawing inappropriate conclusions (e.g., outcome attribution failure).

**HOW TO GET FUNDED:**

• Build the follow-up period into the original proposal if possible.
• Look for funding opportunities that explicitly include a sustainability component – either in the primary grant or through an allowable extension.
• For post hoc-designed analyses, look for small, rapid-response grants.
• Begin to routinize follow-up measurement as the responsibility of local stakeholders.

Before a clear method for measuring the persistence of change can be derived, we believe that there is a critical need for the following:

• A wider use of FE in implementation studies that would better inform measurement of post-implementation sustainability success or failure,

• Availability of a wider array of instruments that measure the important components of sustainability, and

• Inclusion of follow-up analyses built into an original implementation design as a funding expectation.

Until these come about, evaluation of the period after implementation will remain largely relegated to the "need for further research" in most project write-ups. Ultimately, a fuller elucidation of whether quality improvements become institutionalized is needed to determine whether subsidizing implementation of QI yields a sufficient return on the funders' investments.

## Competing interests

The authors declare that they have no competing interests.

## Authors' contributions

CB designed and led this supplemental sustainability analysis and was the predominant contributor to this article. ES was the primary contributor to sections regarding behavioral and organizational factors that influence implementation sustainability. SA and AG are co-directors of QUERI-HIV/Hepatitis and, therefore, oversee all of its projects and publications. SA was the Principal Investigator of this implementation project. All authors read and approved the final manuscript.

## References

[B1] Ovretveit J, Gustafson D (2002). Evaluation of quality improvement programmes. Qual Saf Health Care.

[B2] McQueen L, Mittman BS, Demakis JG (2004). Overview of the Veterans Health Administration (VHA) Quality Enhancement Research Initiative (QUERI). J Am Med Inform Assoc.

[B3] Demakis JG, McQueen L, Kizer KW, Feussner JR (2000). Quality Enhancement Research Initiative (QUERI): A collaboration between research and clinical practice. Med Care.

[B4] Stetler CB, Mittman BS, Francis J (2008). Overview of the VA Quality Enhancement Research Initiative (QUERI) and QUERI theme articles: QUERI Series. Implement Sci.

[B5] Anaya H, B (2003). Results of a national comparative HIV quality-improvement initiative within the VA healthcare system.. 5th International Conference on the Scientific Basis of Health Services: Global Evidence for Local Decisions.

[B6] Greenhalgh T, Robert G, Macfarlane F, Bate P, Kyriakidou O (2004). Diffusion of innovations in service organizations: systematic review and recommendations. Milbank Q.

[B7] Fixsen D, Naoom S, Blase K, Friedman R, Wallace F (2005). Implementation Research: A Synthesis of the Literature..

[B8] Ovretveit J (2003). Making temporary quality improvement continuous. Stockholm: Swedish Association of County Councils.

[B9] LaPelle NR, Zapka J, Ockene JK (2006). Sustainability of public health programs: the example of tobacco treatment services in Massachusetts. Am J Public Health.

[B10] Glasgow RE, Vogt TM, Boles SM (1999). Evaluating the public health impact of health promotion interventions: the RE-AIM framework. Am J Public Health.

[B11] Glasgow RE, Lichtenstein E, Marcus AC (2003). Why don't we see more translation of health promotion research to practice? Rethinking the efficacy-to-effectiveness transition. Am J Public Health.

[B12] Goodman RM, McLeroy KR, Steckler AB, Hoyle RH (1993). Development of level of institutionalization scales for health promotion programs. Health Educ Q.

[B13] Johnson K, Hays C, Center H, Daley C (2004). Building capacity and sustainable prevention innovations: a sustainability planning model.. Evaluation and Program Planning.

[B14] May C, Harrison R, Finch T, MacFarlane A, Mair F, Wallace P (2003). Understanding the normalization of telemedicine services through qualitative evaluation. J Am Med Inform Assoc.

[B15] Klein K, JS S (1996). The challenge of innovation implementation.. Acad Manag Rev.

[B16] Stetler CB, Legro MW, Wallace CM, Bowman C, Guihan M, Hagedorn H, Kimmel B, Sharp ND, Smith JL (2006). The role of formative evaluation in implementation research and the QUERI experience. J Gen Intern Med.

[B17] August GJ, Bloomquist ML, Lee SS, Realmuto GM, Hektner JM (2006). Can evidence-based prevention programs be sustained in community practice settings? The Early Risers' Advanced-Stage Effectiveness Trial. Prev Sci.

[B18] Racine DP (2006). Reliable effectiveness: a theory on sustaining and replicating worthwhile innovations. Adm Policy Ment Health.

[B19] Rosenheck R (2001). Stages in the implementation of innovative clinical programs in complex organizations. J Nerv Ment Dis.

[B20] Carvalho S, White H (2004). Theory-based evaluation: The case of social funds.. Am J Evaluation.

[B21] Green LW (2001). From research to "best practices" in other settings and populations. Am J Health Behav.

[B22] Berwick DM (2003). Disseminating innovations in health care. JAMA.

[B23] Plsek PE, Wilson T (2001). Complexity, leadership, and management in healthcare organisations. BMJ.

[B24] Mant J (2001). Process versus outcome indicators in the assessment of quality of health care. Int J Qual Health Care.

[B25] Goodman RM, Steckler AB (1987). A model for the institutionalization of health promotion programs.. Family & Community Health.

[B26] Orleans CT (2000). Promoting the maintenance of health behavior change: recommendations for the next generation of research and practice. Health Psychol.

[B27] Janz NK, Becker MH (1984). The Health Belief Model: a decade later. Health Educ Q.

[B28] Ajzen I, Fishbein M (1980). Understanding attutudes and predicting social behavior.

[B29] Sobo E (1995). Choosing Unsafe Sex: AIDS-risk Denial among Disadvantaged Women.

[B30] Plsek PE (2003). Complexity and the Adoption of Innovation in Health Care.. Accelerating Quality Improvement in Health Care Strategies to Speed the Diffusion of Evidence-Based Innovations.

[B31] Pluye P, Potvin L, Denis JL, Pelletier J (2004). Program sustainability: focus on organizational routines. Health Promot Int.

[B32] Sobo EJ, Sadler EL (2002). Improving Organizational Communication and Cohesion in a Health Care Setting through Employee-Leadership Exchange. Human Org.

[B33] Sullivan G, Duan N, Mukherjee S, Kirchner J, Perry D, Henderson K (2005). The role of services researchers in facilitating intervention research. Psychiatr Serv.

[B34] Bartholomew L, Parcel G, Kok G, Gottlieb N (2006). Planning health promotion programs: An intervention mapping approach.

[B35] Solberg LI, Brekke ML, Fazio CJ, Fowles J, Jacobsen DN, Kottke TE, Mosser G, O'Connor PJ, Ohnsorg KA, Rolnick SJ (2000). Lessons from experienced guideline implementers: attend to many factors and use multiple strategies. Jt Comm J Qual Improv.

[B36] Wheeler DJ (2004). Advanced Topics in Statistical Process Control.

[B37] Linnan L, Steckler AB and Linnan L (2002). Process evaluation for public health interventions and research: An overview.. Process Evaluation for Public Health Interventions and Research.

[B38] Ferlie EB, Shortell SM (2001). Improving the quality of health care in the United Kingdom and the United States: a framework for change. Milbank Q.

[B39] Fremont AM, Joyce G, Anaya HD, Bowman CC, Halloran JP, Chang SW, Bozzette SA, Asch SM (2006). An HIV collaborative in the VHA: do advanced HIT and one-day sessions change the collaborative experience?. Jt Comm J Qual Patient Saf.

[B40] Titler MG (2004). Methods in translation science. Worldviews Evid Based Nurs.

[B41] Shortell SM, O'Brien JL, Carman JM, Foster RW, Hughes EF, Boerstler H, O'Connor EJ (1995). Assessing the impact of continuous quality improvement/total quality management: concept versus implementation. Health Serv Res.

[B42] Harvey G, Wensing M (2003). Methods for evaluation of small scale quality improvement projects. Qual Saf Health Care.

[B43] Ovretveit J, Bate P, Cleary P, Cretin S, Gustafson D, McInnes K, McLeod H, Molfenter T, Plsek P, Robert G, Shortell S, Wilson T (2002). Quality collaboratives: lessons from research. Qual Saf Health Care.

[B44] Shojania KG, Grimshaw JM (2005). Evidence-based quality improvement: the state of the science. Health Aff (Millwood ).

[B45] Patterson ES, Nguyen AD, Halloran JP, Asch SM (2004). Human factors barriers to the effective use of ten HIV clinical reminders. J Am Med Inform Assoc.

[B46] Knox KL, Litts DA, Talcott GW, Feig JC, Caine ED (2003). Risk of suicide and related adverse outcomes after exposure to a suicide prevention programme in the US Air Force: cohort study. BMJ.

[B47] Harland J, White M, Drinkwater C, Chinn D, Farr L, Howel D (1999). The Newcastle exercise project: a randomised controlled trial of methods to promote physical activity in primary care. BMJ.

[B48] Shye D, Feldman V, Hokanson CS, Mullooly JP (2004). Secondary prevention of domestic violence in HMO primary care: evaluation of alternative implementation strategies. Am J Manag Care.

[B49] Sanci LA, Coffey CM, Veit FC, Carr-Gregg M, Patton GC, Day N, Bowes G (2000). Evaluation of the effectiveness of an educational intervention for general practitioners in adolescent health care: randomised controlled trial. BMJ.

[B50] Sanci L, Coffey C, Patton G, Bowes G (2005). Sustainability of change with quality general practitioner education in adolescent health: a 5-year follow-up. Med Educ.

[B51] Perlstein PH, Kotagal UR, Schoettker PJ, Atherton HD, Farrell MK, Gerhardt WE, Alfaro MP (2000). Sustaining the implementation of an evidence-based guideline for bronchiolitis. Arch Pediatr Adolesc Med.

[B52] Brand C, Landgren F, Hutchinson A, Jones C, Macgregor L, Campbell D (2005). Clinical practice guidelines: barriers to durability after effective early implementation. Intern Med J.

[B53] Morgenstern LB, Bartholomew LK, Grotta JC, Staub L, King M, Chan W (2003). Sustained benefit of a community and professional intervention to increase acute stroke therapy. Arch Intern Med.

[B54] Bere E, Veierod MB, Bjelland M, Klepp KI (2006). Free school fruit--sustained effect 1 year later. Health Educ Res.

[B55] Shepherd J, Peersman G, Weston R, Napuli I (2000). Cervical cancer and sexual lifestyle: a systematic review of health education interventions targeted at women. Health Educ Res.

